# Supplementary material for: Computer-aided identification of degenerative neuromuscular diseases based on gait dynamics and ensemble decision tree classifiers
Source: PLoS One. 2021 Jun 4;16(6):e0252380. doi: 10.1371/journal.pone.0252380 (PMC8177554; doi:10.1371/journal.pone.0252380)
Supplement: Attachment — Submitted filename: Response to Reviewers.docx [file pone.0252380.s001.docx]

**Manuscript Number:** PONE-D-21-08939

**Manuscript Title:** Computer-aided identification of degenerative neuromuscular diseases based on gait dynamics and ensemble decision tree classifiers

**To:** PLOS ONE

**Re:** PLOS ONE Decision: Revision required [PONE-D-21-08939] – [EMID:073fa78878e4ee52]

Dear Editor,

Thank you for your email enclosing the reviewers’ comments. We appreciate this valuable input, which resulted in improving the quality of the manuscript.

We have carefully reviewed all the comments and revised the manuscript accordingly. We are uploading:

- Our point-by-point response to the reviewers (below)
- An updated manuscript with changes highlighted in blue (labeled: Revised Manuscript with Track Changes)
- An unmarked version of your revised paper without tracked changes (labeled: Manuscript).

Thank you for allowing the resubmission of our manuscript. We hope the revised version is now suitable for publication and look forward to hearing from you soon.

Sincerely,

Luay Fraiwan et al.

Reviewer 1

**Comment 1:** **No need of making separate subsections 1.1 and 1.2.**

**Response:** We thank the reviewer for this recommendation. The “contributions” and “paper organization” subsections have been integrated with the introduction section for a more coherent flow of ideas.

**Edits:** Please note the removed headings of subsections 1.1 and 1.2.

**Comment 2:** **Where is the related work section? Limitations in existing work should lead to the proposed work section.**

**Response:** We thank the reviewer for this comment. We have integrated the related work section within the introduction (please see lines 42-71). In response to the reviewer's comment, we have discussed the limitations of previous works in relation to the contributions of this study.

**Edits:** Please note the added paragraph after the literature review to discuss the limitations of previous works according to the following:

“According to previous studies, deep learning-based models tend to exhibit a highly auspicious performance when classifying DNDs in both binary and multiclass contexts. In most cases, complicated preprocessing and feature engineering techniques were also used. Compared to traditional machine learning and pattern recognition methods, training and validating a reliable deep learning architecture requires significant computational resources. Typically, this process is iterative, involves multiple model parameters, and entails specialized graphical processing units. The lack of sufficient, high-quality, and comprehensive clinical data is also considered amongst the main limitations. To exploit the value of automated disease detection systems in resource-constrained settings where only small datasets and low-cost hardware devices are available, simplistic computational approaches to characterize and classify gait patterns are worth investigation.”

**Comment 3:** **Authors can consider the following recent references:**

1 Long-term Wind Power Forecasting using Tree-based Learning Algorithms.
2 A Consolidated Decision Tree-based Intrusion Detection System for binary and multiclass imbalanced datasets.
3 A Trusted Social Network using Hypothetical Mathematical Model and Decision-based Scheme.

**Response:** We thank the reviewer for the suggested interesting articles. However, we believe that their scope and objectives are not entirely pertinent to our study. Moreover, even though studies 1 and 2 used decision tree-based models for classification, only a basic background explanation was provided without mathematical or implementation details on the algorithms employed to construct the models. Therefore, we cannot decide if these studies used the same implementations used in this work.

**Comment 4:** **Discussion and Conclusion should not be clubbed.**

**Response:** We thank the reviewer for this suggestion. Please note the added conclusion section.

**Edits:**

“**Conclusion and Future work**

This paper investigates the application of ensemble classification to identify different DNDs. Based on normal-paced gait fluctuations, healthy and disease conditions were characterized using spatiotemporal statistical features derived from VGRF signals. For the classification task, several ensemble classification approaches were investigated based on a base decision tree classifier. A data-driven hyperparameter tuning approach using Bayesian optimization was employed to select the most proper parameter for all classification methods. The obtained results demonstrated the promising capability of detecting common neurodegenerative diseases, with the highest overall classification rate of 99.17%. Thus, the proposed framework is applicable to aid in diagnostic decisions while considering computing hardware resource-restricted environments. This framework can be extended in future work to include other types of DNDs and spatiotemporal gait patterns. However, this requires further experimentation spanning a broader range of subjects and disease conditions. Moreover, the investigation of other feature extraction approaches and deep learning classification models is expected to improve classification performance.”

**Comment 5:** **It is always necessary to analyze the results rather than discussing only.**

**Response:** We thank the reviewer for this comment. We have analyzed our key finding and observations with more details in the discussion section.

**Edits:** Please not the edits made to the discussion according to the following. Edits relative to the original text are indicated in blue.

“The prospect of machine learning usually requires extensive data transformations to provide the best possible training set to the learner. Amongst the main aspects related to data transformation is feature extraction. Optimal feature extraction provides a better representation of patterns under investigation and improves the models’ predictive performance. In our proposed framework, gait signals were characterized based on statistical features, including the RMS, variance, kurtosis, and skewness. One of the main strengths of the proposed framework is using a limited number of simple features to characterize gait dynamics. These features were derived directly from raw and short-length gait singles without applying extensive preprocessing or complex filtering or transformation techniques. Such characteristic adds to the computational efficiency of the proposed framework and facilitates its application in real-time settings. Despite the simplicity, our statistical analysis showed that these features positively represented characteristic variations between disease groups. Post-hoc comparisons revealed that the features derived from the raw VGRF signals corresponded to more significant pair-wise group differences.

For DND detection, we employed four types of ensemble classifiers: bagging, AdaBoost, RUSBoost, and random subspace. In order to highlight the significance of ensembled predictions, we also considered the performance of the base decision tree model. In line with the statistical analysis results, the classification analysis showed that the models’ predictive performance was influenced by variability in the gait feature. Evaluation of classification performance further emphasized that the VGRF-based feature set exhibited a notably higher predictive efficiency than the other three feature sets, regardless of the classification model used. Our target of achieving a high-performance detection framework was accomplished using the AdaBoost classifier in conjugation with the VGRF-based feature set, with an average classification accuracy of 99.17%. Correspondingly, the class-specific accuracies of 98.8, 98.8%, 98.8%, and 99.4% were achieved for the control, ALS, PD, and HD groups, respectively. Similarly, using the features extracted from the gait parameter signals, the AdaBoost model generally provided superior performance. However, we obtained a lower overall accuracy of 81.98% for the stance-based feature set, 79.68% for the stride-based feature set, and 79.30% for the swing-based feature set.

Worth noting, the classification results provided empirical evidence suggesting that ensemble classifier systems are better performers than their constituent base models. Using the base decision tree model , the VGRF-based features set provided the best classification performance, but ultimately, all ensemble techniques improved classification results to varying degrees of success. The AdaBoost yielded the most considerable improvement in all metrics, with an improvement percentage of 6.49%, 13.74%, 4.26%, 14.32%, 20.02% for the accuracy, sensitivity, specificity, F1-score, and Cohen’s kappa coefficient, respectively. Following Adaboost, random subspace performed best with a 4.48% increment in the accuracy, 9.14% in the sensitivity, and 2.89% in the specificity. Slightly lower performance improvements were associated with bagging and RUSBoosting models. For the gait parameter signals, the AdaBoost model showed the most notable performance improvement. The associated percentage increase in detection accuracy, sensitivity, and specificity ranged between 0.78%-2.72%, 2.14%-8.93%, and 0.61%-1.64%, respectively.

The physionet gait database was used in a few recent studies to perform a multi-class classification of neurodegenerative diseases. Table \ref{literature} provides a comparative summary of these works. In agreement with our proposed framework, adopted literature approaches generally integrated a wide range of feature extraction methods with supervised machine learning classification. The feature extraction methods for the VGRF signals included statistical amplitude quantification, detrended fluctuation analysis, and fractal dimension. The features characterizing gait parameter signals were based on statistical amplitude quantification and recurrent analysis. For the classification task, a limited range of standard algorithms was explored, i.e., adaptive boosting trees, random forests (RF), support vector machines, and sparse non-negative least squares (NNLS) coding. Athisakthi et al. reported the highest accuracy for the parameter gait signal through using Wavelet transform-based statistical features and RF classifier (stride 91.75%, stance 93.74%, and swing 93.7%) \cite{M5}. However, the best overall accuracy of 98.45% was obtained through statistical characterization of VGRF signals alongside NNLS coding classification \cite{M2}. Thus, it can be noted that our proposed framework significantly improved the neurodegenerative disease recognition rate in comparison to the state-of-the-art methods in the literature. Potential advantages of such an accurate diagnostic system include aiding in smart long-term monitoring. This also supports clinicians and care providers with noninvasive and low-cost tools to aid in making diagnostic decisions. A possible explanation for the relatively lower accuracies obtained using the stride, stance, and swing parameters signals might be due to the small-length raw VGRF signals used to derive these signals. However, this approach was followed since the available data set is not large enough to perform multiple fold validation.”

**Comment 6:** **Future work should be discussed with more clarity.**

**Response:** We thank the reviewer for this suggestion. Future work has been articulated in the added conclusion section.

**Edits:** Please refer to the edits made in response to comment 4.

**Comment 7:** **Write the text in simple present tense.**

**Response:** We thank the reviewer for this suggestion. However, we would like to remind that this paper was written to report a study done in the past. It thus describes what was done and reports the results obtained. To present the paper in a narrative structure, we used a combination of past and present tenses. We used the past tense for elapsed events and the present tense for general facts and findings as follows:

*Introduction:*

- Background information and facts: simple present tense
- Literature review: simple past and present perfect tense (past events with present consequences)
- Objectives: simple present tense
- Contributions: simple past tense

*Methodology:* simple past tense

*Results:*

- Reporting actual results obtained: simple past tense
- Explaining or referring to tables and figures: simple present tense

*Discussion:*

- summary of findings: simple past tense
- Interpretation of significant findings: simple present tense

*Conclusion:*

- Refer to completed research aspects: simple past tense
- Study implications and findings: simple present tense
- Future research directions: future and simple present tense

Please note that this writing standard is adopted in almost all published research papers. The popular book of [Malcolm A. Moore](https://www.google.ae/search?tbo=p&tbm=bks&q=inauthor:%22Malcolm+A.+Moore%22&source=gbs_metadata_r&cad=2), titled: A Practical Guide to the Use of Scientific English, also recommended the above guidelines related to verb tense usage in a typical research paper.

**Comment 8:** **English editing required.**

**Response:** We thank the reviewer for this note. The paper has been thoroughly proofread to modify grammatical errors and typos.

Reviewer 2

**Comment 1:** **Please improve overall readability of the paper.**

**Response:** The paper has been thoroughly edited and several words/sentences have been substituted or re-phrased appropriately, to improve readability.

**Comment 2:** **I can see some paragraph in introduction, related work and proposed approach which should be merged. Same thing is with background section. Please reduce text and improve the representation of this section.**

**Response:** We thank the reviewer for this comment. The “contributions” and “paper organization” subsections have been integrated with the introduction section for a more coherent flow of ideas. We also removed some redundant and unnecessary information and sentences from the background about degenerative neuromuscular diseases and gait fluctuation analysis.

**Edits:** Please note the removed headings of subsections 1.1 and 1.2. Also, note the updated background of degenerative neuromuscular diseases, as per the following. Please refer to the edits made in comment 4 regarding reducing the text of gait fluctuation analysis.

“Human motion is controlled by the neuromuscular system, which comprises all muscles, sensory neurons, and motor neurons [1]. Degenerative neuromuscular disease (DNDs) arises from the degeneration or progressive loss of the function in efferent or afferent4nerves. Efferent nerves are responsible for controlling voluntary muscles, while afferent nerves communicate sensory information back to the brain and the central nervous6system [2]. Examples of common DNDs include amyotrophic lateral sclerosis (ALS), Parkinson’s disease (PD), and Huntington’s disease (HD). ALS is a progressive condition attributed to the preferential degeneration of upper and lower motor neurons [3, 4]. As such, the disease impacts nerve cells controlling voluntary muscle control, leading to a debilitated state affecting breathing, motion, speech, eating, and even cognition [5]. PD is caused by neuron loss in the substantia nigra, a structure responsible for releasing the neurotransmitter dopamine and plays a vital role in learning, reward, and movement [6]. PD is often associated with motor symptoms, including muscle rigidity, posture instability, rhythmic resting tremors, bradykinesia, and gait festination, propulsion, and freezing [7]. HD is a genetic condition that also16affects the basal ganglia and occurs explicitly due to the loss of spiny projection neurons [3]. HD’s main characteristic symptom is hyperkinesia, a state of excessive restlessness leading to involuntary chorea movements. Other symptoms May include cognitive degeneration and psychiatric dysfunction [8, 9].“

**Comment 3:** **The objectives of this paper need to be polished. Contribution list should be polished at the end of the introduction section and last paragraph of the introduction should be the organization of the paper.**

**Response:** We thank the reviewer for this comment. We have improved the representation of this part by first stating the main objectives of this paper followed by the contributions made to achieve these objectives. The paper organization is outlined next as the last paragraph in the introduction.

**Edits:** Please note the edits made to the study objectives and contributions according to the following:

“To address the shortcomings of previous works, this study proposes a simple yet reliable computer-aided framework that simultaneously detects a wide range of DNDs based on gait dynamics. Our primary objective is to perform a comparative performance investigation for different combinations of spatiotemporal gait patterns and ensemble classification methods. To this end, we first proposed a new approach to derive spatiotemporal gait cycle time series from VGRF signals. This approach was applied to derive parameters such as stride duration, stance duration, and swing duration. Feature characterization of the VGRF signals and the spatiotemporal gait signals was based on the statistical descriptors of root mean square, variance, skewness, and kurtosis. These descriptors were applied to raw short-length signals to maximize data availability and support the proposed framework's computational efficiency. Finally, we compared the performance of various DT ensemble models based on the concepts of bagging, adaptive boosting (AdaBoost), random under-sampling boosting (RUSBoost), and random subspace. Fig. 1 illustrates the DNDs detection framework employed in this study.”

**Comment 4:** **Introduction is poorly written. Please add background of gait fluctuations studies in the introduction.**

**Response:** We thank the reviewer for this comment. The entire introduction section has been edited, as per comments 2,3,4, 5, and 6. We have also added some background about gait fluctuation analysis and the studies relating it to degenerative neuromuscular diseases.

**Edits:** Please not the added background about gait fluctuation analysis, as pe the following. Edits relative to the original text are indicated in blue.

“Studying human locomotion to diagnose DNDs shows great promise [10]. The study of human locomotion is traditionally performed using gait fluctuation analysis and aims to extract useful spatial and temporal information quantifying human motion [11]. The data recorded using typical gait measurement systems are of periodic nature. A single gait cycle consists of a sequence of spatial events attributed to the timely foot-floor contact activity. These events, namely stride, stance, and swing, can be marked vertical ground reaction force (VGRF) signals. Typically investigated temporal attributes of gait cycle events include duration and rate. DNDs can pose significant locomotion abnormalities, reflecting on the associated gait patterns during normal walking. Accumulated studies have shown that these abnormalities are disease-specific, and thus, gait analysis can be an effective tool to differentiate and diagnose DNDs [10, 12]. For example, Ren et al. [13] used phase synchronization and conditional entropy as parameters to distinguish healthy subjects and subjects with three neurodegenerative diseases: PD, ALS, and HD. These two parameters were calculated for five pairs of time series rhythms: stance time, swing time, stride time, percentage of swing time, and stance time percentage. Another work was done by Jian-Jun et al. [14] where the Hurst exponent was used as an indicator of aging and neurodegenerative diseases. They found that the Hurt exponent of stride intervals decreases with neurodegenerative diseases and aging. In accordance, Huasdorff et al. [15] reported a significant correlation between stride interval, aging, and HD. Older subjects and HD patients had reduced stride intervals compared to healthy subjects.“

**Comment 5:** **Contributions at the end of introduction section should be polished.**

**Response:** We thank the reviewer for this comment. We have improved the representation of this part by first stating the main objectives of this paper followed by the contributions made to achieve these objectives.

**Edits:** Please note the edits made in response to comment 3.

**Comment 6:** **Relevant literature review of latest similar research studies on the topic at hand must be discussed.**

**Response:** We thank the reviewer for this comment. we improved the representation of the literature review in the introduction section by rewriting the related works paragraph in a more concise and readable manner. Also, we added more recent references relevant to this study. Moreover, the limitations of previous works were polished to further highlight the contribution of our work.

**Edits:** Please note the updated literature review in the introduction section as per the following:

“Driven by the need for economic non-invasive clinical practices, the application of computer-aided human locomotion analysis to diagnose DNDs has recently gained significant research traction. Computer-aided diagnostic systems typically integrate artificial intelligence algorithms. If the data is obtained and processed appropriately, and the detection algorithm is well chosen and optimized, the elements of human expertise and error become less detrimental to the diagnosis process. On these grounds,47an extensive class of previous studies was directed towards the binary classification of normal vs. pathological conditions [16–23] . Standard procedures extracted features included statistics values [16–20], recurrence quantification analysis parameters [17], fuzzy recurrence plots [18], topological motion analysis [21, 22], and left/right-foot autocorrelation and cross correlation [23]. Employed machine learning and deep learning methods included support vector machine (SVM) [16–18], least squares SVM (LS-SVM) [18], k-nearest neighbors (KNN) [16, 21], naive Bayes [21], random forest (RF) [22], decision trees [23], adaptive Neuro-Fuzzy Inference [20], multi-layer perceptron (MLP) [16], probabilistic neural network (PNN) [17], and convolutional neural network (CNN) [19].

Worth noting, the three types of DNDs discussed earlier share over-lapping motor symptoms. Thus, as targeted in this study, an efficient approach would be needed to classify these conditions simultaneously. In accordance, a limited number of recent studies tackled multiclass classification of DNDs. In [24], Beyrami et al. used a wide range of statistical and entropy features alongside a non-negative least squares (NNLS) classifier. This approach was applied to raw short-length VGRF signals only. Lin C-W et al. [25] investigated recurrence plot and principal component analysis to transform time-domain VGRF signals into images. These images were inputted as features to a CNN model for classification. On the contrary, Alaska et al. [26] compared the performance of several classification models, namely artificial neural network (ANN), KNN, linear SVM, and RF. In the feature transformation process, extracted temporal68and spectral features included independent reconstruction components, approximate entropy, standard deviation, minimum, maximum, and mean values, and the ratio of peak-magnitude to root-mean-square.”

**Comment 7**: Quality of figures are not good.

**Response:** We thank the reviewer for this observation. Figures’ quality has been improved by increasing font size and rearranging some of the elements. Moreover, the figures have been saved in pdf format instead of tiff format to maintain their high resolution.

**Comment 8:** **Result section need to be polished.**

**Response:** We thank the reviewer for this suggestion. We have improved the representation and text of the classification analysis results.

**Edits:** Please not the edits made to the classification results section according to the following:

**3.2 Classification Results**

Table 3 compares the performance of the investigated classification models as applied to the features derived from the VGRF, stride, stance, and swing signals independently. The tabulated values represent the average of the classification accuracy, sensitivity, specificity, F-score, and Cohen's kappa coefficient over validation folds.

**3.2.1 VGRF-Based Features**

Using the statistical features derived from the left and right VGRF signals, the results show that the base DT model performed poorly compared to the other ensemble classifiers with an overall average accuracy of 93.13%, sensitivity of 98.23%, specificity of 99.43%, F1-Score of 85.97% and Cohen’s kappa coefficient of 81.43%. Using the AdaBoost ensemble approach, the overall performance notably improved, providing an average classification accuracy of 99.17%. Using the same classification model, the sensitivity, specificity, F1-score, and Cohen’s kappa coefficient metrics reached 99.17%, 98.23%, 99.43%, 98.28%, and 97.73%, respectively. Slightly lower classification accuracies were observed for the random subspace (97.30%), Bagging (96.57%), and Boosting (96.66%) ensembles.

**3.2.2 Stride-Based Features**

As shown in Table 3, the AdaBoost model provided the highest detection accuracy for the stride-based feature set at an overall average of 97.68%. The DT and Random Subspace models a relatively lower classification accuracy of 79.06%. The highest sensitivity (58.65%), specificity (86.26%), F1-Score (58.10%) and Cohen’s kappa coefficient (44.60%) were also obtained by the AdaBoost classifier. On the contrary, the Bagging ensemble model provided the worst performance, as demonstrated by its classification accuracy of 78.53%. All other metrics dropped to 56.31%, 85.34%, 55.17%, and 41.09% for the sensitivity, specificity, F1-score, and Cohen’s kappa coefficients, respectively.

**3.2.3 Stance-Based Features**

The best classification performance for the stance-based feature set was attained using the AdaBoost classifier at an accuracy of 81.98%. Concurrently, the highest sensitivity of 63.54%, specificity of 87.81%, F1-score of 63.18% and Cohen’s kappa coefficient of 51.18% were obtained using the same model. The Random substance ensemble performed second to AdaBoost, followed by RUSBoost, then Bagging ensembles with average accuracies ranging between 80.96%-80.35%. On the contrary, the base DT model provided the worst performance as evidenced by its accuracy (80.13%), sensitivity (59.60%), specificity (86.57%), F1-score (59.54%), and Cohen’s kappa coefficient (46.37%).

**3.2.4 Swing-Based Features**

The swing-based features displayed a similar pattern to that obtained using the stride and stance features. The AdaBoost model provided a superior overall performance with an accuracy of 79.30%, sensitivity of 55.97%, specificity of 85.71%, F1-score of 56.19%, and Cohen’s kappa coefficient of 42.51%. Following, the RUSBoost and random subspace ensembles demonstrated a slightly worse performance with overall overage accuracy of 77.73% and 77.71%, respectively. The worst performance among all classification models and feature sets was obtained using the bagging ensemble as evidenced by the obtained accuracy (77.20%), sensitivity (49.56%), specificity (84.08%), F1-score (47.42%), and Cohen’s kappa coefficient (33.38%).

**Comment 9:** **All abbreviations must be described.**

**Response:** We thank the reviewer for this note. For the abstract section, the significant terms were introduced with their abbreviation at first occurrence. Starting from the introduction section and throughout the whole manuscript, all abbreviations were introduced only at the first occurrence in the text. In figures, all abbreviations were described within the caption. For tables, abbreviations were described in a note underneath the table.

**Comment 10:** **There are some grammar and typo errors.**

**Response**: We thank the reviewer for this not. The paper has been thoroughly proofread to modify grammatical errors and typos.

**Comment 11:** Authors must add more up to date references related to security of 2020,2021 in introduction and literature.

**Response:** We thank the reviewer for this suggestion. The introduction and literature have been updated with recent references.

**Edits:** The following recent references have been incorporated within the introduction and literature review.

- Dadar M, Manera AL, Zinman L, Korngut L, Genge A, Graham SJ, et al. Cerebral atrophy in amyotrophic lateral sclerosis parallels the pathological distribution of TDP43. Brain Communications. 2020;2(2).
- Prabhu P, Karunakar AK, Anitha H, Pradhan N. Classification of gait signals into different neurodegenerative diseases using statistical analysis and recurrence quantification analysis. Pattern Recognition Letters. 2020;139:10–16.
- Lin CW, Wen TC, Setiawan F. Evaluation of Vertical Ground Reaction Forces Pattern Visualization in Neurodegenerative Diseases Identification Using Deep Learning and Recurrence Plot Image Feature Extraction. Sensors. 2020;20(14).
- Yan Y, Ivanov K, Mumini Omisore O, Igbe T, Liu Q, Nie Z, et al. Gait Rhythm Dynamics for Neuro-Degenerative Disease Classification via Persistence Landscape- Based Topological Representation. Sensors. 2020;20(7):1-24.
- Yan Y, Omisore OM, Xue Y, Li H, Liu Q, Nie Z, et al. Classification of Neurodegenerative Diseases via Topological Motion Analysis—A Comparison Study for Multiple Gait Fluctuations. IEEE Access. 2020;8:96363–96377.
- Marziyeh Ghoreshi Beyrami S, Ghaderyan P. A robust, cost-effective and non-invasive computer-aided method for diagnosis three types of neurodegenerative diseases with gait signal analysis. Measurement. 2020;156:1–15.
- Haya A, Hussain A, Khan W, Tawfik H, Trevorrow P, Liatsis P, et al. A data science approach for reliable classification of neuro-degenerative diseases using gait patterns. Journal of Reliable Intelligent Environments. 2020; p. 233–247.
- Lin CW, Wen TC, Setiawan F. Evaluation of Vertical Ground Reaction Forces Pattern Visualization in Neurodegenerative Diseases Identification Using Deep Learning and Recurrence Plot Image Feature Extraction. Sensors. 2020;20(14).

**Comment 12:** **Authors should add these recent references:**

1 BCD-WERT: a novel approach for breast cancer detection using whale optimization based efficient features and extremely randomized tree algorithm, peerj computer science
2 Automated cognitive health assessment in smart homes using machine learning, Sustainable Cities and Society.

**Response:** We thank the reviewer for suggesting these interesting articles. However, we believe that their scope and objectives are not entirely pertinent to our study. Even though study 1 used a decision tree model for classification, only a basic background explanation was provided. No mathematical or implementation details on the algorithms employed to construct the model were provided. Moreover, study 1 employed the information gain as the node split criterion. In this work, the CART algorithm was employed to construct the decision models at the training stage, and the Gini's diversity index was employed as the root node split criterion. Therefore, the decision tree model in this study had a different implementation to the base decision tree model used in this work.

As for study 2, the AdaBoost model matched our implementation, according to the provided pseudo code (Algorithm 5) and equations. Therefore, this study was cited in our classification models' methodology.

**Edits:** Please not the cited study in section 2.3 according to the following text:

“The mathematical formulation of these classification methods is detailed in [36-40].”

Reviewer 3

**Comment 1:** **Summarize the related works in the form of a table.**

**Response:** We thank the reviewer for this suggestion. However, we would like to point out that the most relevant works in literature were already summarized in a table in the discussion section. The studies included in this tabulated summary were restricted to those explicitly focusing on performing multi-class classification of degenerative neuromuscular diseases using the publicly available PhysioNet data set. These studies were identified after reviewing all the papers citing the PhysioNet Neurodegenerative disease dataset. We believe that keeping such concise representation in the discussion section will facilitate direct comparison to our proposed methodology and obtained results.

In response to the reviewer’s comment, we improved the representation of the literature review in the introduction section by rewriting the related works paragraph in a more concise and readable manner. Also, we added more recent references relevant to this study. Moreover, the limitations of previous works were polished to further highlight the contribution of our work.

**Edits:** Please note the updated literature review in the introduction section as per the following:

“Driven by the need for economic non-invasive clinical practices, the application of computer-aided human locomotion analysis to diagnose DNDs has recently gained significant research traction. Computer-aided diagnostic systems typically integrate artificial intelligence algorithms. If the data is obtained and processed appropriately, and the detection algorithm is well chosen and optimized, the elements of human expertise and error become less detrimental to the diagnosis process. On these grounds,47an extensive class of previous studies was directed towards the binary classification of normal vs. pathological conditions [16–23] . Standard procedures extracted features included statistics values [16–20], recurrence quantification analysis parameters [17], fuzzy recurrence plots [18], topological motion analysis [21, 22], and left/right-foot autocorrelation and cross correlation [23]. Employed machine learning and deep learning methods included support vector machine (SVM) [16–18], least squares SVM (LS-SVM) [18], k-nearest neighbors (KNN) [16, 21], naive Bayes [21], random forest (RF) [22], decision trees [23], adaptive Neuro-Fuzzy Inference [20], multi-layer perceptron (MLP) [16], probabilistic neural network (PNN) [17], and convolutional neural network (CNN) [19].

Worth noting, the three types of DNDs discussed earlier share over-lapping motor symptoms. Thus, as targeted in this study, an efficient approach would be needed to classify these conditions simultaneously. In accordance, a limited number of recent studies tackled multiclass classification of DNDs. In [24], Beyrami et al. used a wide range of statistical and entropy features alongside a non-negative least squares (NNLS) classifier. This approach was applied to raw short-length VGRF signals only. Lin C-W et al. [25] investigated recurrence plot and principal component analysis to transform time-domain VGRF signals into images. These images were inputted as features to a CNN model for classification. On the contrary, Alaska et al. [26] compared the performance of several classification models, namely artificial neural network (ANN), KNN, linear SVM, and RF. In the feature transformation process, extracted temporal68and spectral features included independent reconstruction components, approximate entropy, standard deviation, minimum, maximum, and mean values, and the ratio of peak-magnitude to root-mean-square.”

**Comment 2:** **Some of the recent and relevant works such as the following can be discussed in the paper:**

1 An ensemble machine learning approach through effective feature extraction to classify fake news
2 Early detection of diabetic retinopathy using PCA-firefly based deep learning model
3 An adaptive multi-layer botnet detection technique using machine learning classifiers

**Response:** We thank the reviewer for the suggested interesting articles. However, we believe that their scope and objectives are not entirely pertinent to our study. Moreover, even though these studies used a decision tree model for classification or feature selection purposes, no mathematical or implementation details were provided (i.e. the algorithm employed to construct the model and the root node split criterion). For example, some of the notable DT implementations include classification and regression tree (CART), chi-square automatic interaction detection (CHAID), and iterative dichotomiser 3 (ID3). Popular node split criteria indices include Gini diversity, information gain, and chi-square. In this work, the CART algorithm was employed to construct the DT models at the training stage, and the Gini's diversity index was employed as the root node split criterion. Therefore, we cannot decide if the implemented decision tree models in these studies had the exact implementation to the base decision tree model used in this work.

**Comment 3:** **Present a detailed analysis on the results obtained.**

**Response:** We thank the reviewer for this comment. We have analyzed our key finding and observations with more details in the discussion section.

**Edits:** Please not the edits made to the discussion according to the following. Edits relative to the original text are indicated in blue.

“The prospect of machine learning usually requires extensive data transformations to provide the best possible training set to the learner. Amongst the main aspects related to data transformation is feature extraction. Optimal feature extraction provides a better representation of patterns under investigation and improves the models’ predictive performance. In our proposed framework, gait signals were characterized based on statistical features, including the RMS, variance, kurtosis, and skewness. One of the main strengths of the proposed framework is using a limited number of simple features to characterize gait dynamics. These features were derived directly from raw and short-length gait singles without applying extensive preprocessing or complex filtering or transformation techniques. Such characteristic adds to the computational efficiency of the proposed framework and facilitates its application in real-time settings. Despite the simplicity, our statistical analysis showed that these features positively represented characteristic variations between disease groups. Post-hoc comparisons revealed that the features derived from the raw VGRF signals corresponded to more significant pair-wise group differences.

For DND detection, we employed four types of ensemble classifiers: bagging, AdaBoost, RUSBoost, and random subspace. In order to highlight the significance of ensembled predictions, we also considered the performance of the base decision tree model. In line with the statistical analysis results, the classification analysis showed that the models’ predictive performance was influenced by variability in the gait feature. Evaluation of classification performance further emphasized that the VGRF-based feature set exhibited a notably higher predictive efficiency than the other three feature sets, regardless of the classification model used. Our target of achieving a high-performance detection framework was accomplished using the AdaBoost classifier in conjugation with the VGRF-based feature set, with an average classification accuracy of 99.17%. Correspondingly, the class-specific accuracies of 98.8, 98.8%, 98.8%, and 99.4% were achieved for the control, ALS, PD, and HD groups, respectively. Similarly, using the features extracted from the gait parameter signals, the AdaBoost model generally provided superior performance. However, we obtained a lower overall accuracy of 81.98% for the stance-based feature set, 79.68% for the stride-based feature set, and 79.30% for the swing-based feature set.

Worth noting, the classification results provided empirical evidence suggesting that ensemble classifier systems are better performers than their constituent base models. Using the base decision tree model , the VGRF-based features set provided the best classification performance, but ultimately, all ensemble techniques improved classification results to varying degrees of success. The AdaBoost yielded the most considerable improvement in all metrics, with an improvement percentage of 6.49%, 13.74%, 4.26%, 14.32%, 20.02% for the accuracy, sensitivity, specificity, F1-score, and Cohen’s kappa coefficient, respectively. Following Adaboost, random subspace performed best with a 4.48% increment in the accuracy, 9.14% in the sensitivity, and 2.89% in the specificity. Slightly lower performance improvements were associated with bagging and RUSBoosting models. For the gait parameter signals, the AdaBoost model showed the most notable performance improvement. The associated percentage increase in detection accuracy, sensitivity, and specificity ranged between 0.78%-2.72%, 2.14%-8.93%, and 0.61%-1.64%, respectively.

The physionet gait database was used in a few recent studies to perform a multi-class classification of neurodegenerative diseases. Table \ref{literature} provides a comparative summary of these works. In agreement with our proposed framework, adopted literature approaches generally integrated a wide range of feature extraction methods with supervised machine learning classification. The feature extraction methods for the VGRF signals included statistical amplitude quantification, detrended fluctuation analysis, and fractal dimension. The features characterizing gait parameter signals were based on statistical amplitude quantification and recurrent analysis. For the classification task, a limited range of standard algorithms was explored, i.e., adaptive boosting trees, random forests (RF), support vector machines, and sparse non-negative least squares (NNLS) coding. Athisakthi et al. reported the highest accuracy for the parameter gait signal through using Wavelet transform-based statistical features and RF classifier (stride 91.75%, stance 93.74%, and swing 93.7%) \cite{M5}. However, the best overall accuracy of 98.45% was obtained through statistical characterization of VGRF signals alongside NNLS coding classification \cite{M2}. Thus, it can be noted that our proposed framework significantly improved the neurodegenerative disease recognition rate in comparison to the state-of-the-art methods in the literature. Potential advantages of such an accurate diagnostic system include aiding in smart long-term monitoring. This also supports clinicians and care providers with noninvasive and low-cost tools to aid in making diagnostic decisions. A possible explanation for the relatively lower accuracies obtained using the stride, stance, and swing parameters signals might be due to the small-length raw VGRF signals used to derive these signals. However, this approach was followed since the available data set is not large enough to perform multiple fold validation.”

**Comment 4:** **Discuss about the limitations and future scope of the current work.**

**Response:** We thank the reviewer for this suggestion. We have discussed the limitations of this study at the end of the discussion section. Also, we have added a separate conclusion section (section 5) highlighting future research directions.

**Edits:**

**Study limitations**

“There may exist several methodological limitations in this study. Patient-specific factors such as age, gender, and disease severity were incongruent between different disease groups. Inconsistencies in such subject-specific factors could have a direct effect on the classification model's predictive performance. The availability of a more comprehensive data set is essential to investigate the impact of these factors and, therefore, support the generalization ability of the proposed framework.”

**Conclusion and Future work**

“This paper investigates the application of ensemble classification to identify different DNDs. Based on normal-paced gait fluctuations, healthy and disease conditions were characterized using spatiotemporal statistical features derived from VGRF signals. For the classification task, several ensemble classification approaches were investigated based on a base decision tree classifier. A data-driven hyperparameter tuning approach using Bayesian optimization was employed to select the most proper parameter for all classification methods. The obtained results demonstrated the promising capability of detecting common neurodegenerative diseases, with the highest overall classification rate of 99.17%. Thus, the proposed framework is applicable to aid in diagnostic decisions while considering computing hardware resource-restricted environments. This framework can be extended in future work to include other types of DNDs and spatiotemporal gait patterns. However, this requires further experimentation spanning a broader range of subjects and disease conditions. Moreover, the investigation of other feature extraction approaches and deep learning classification models is expected to improve classification performance.”
